# Comparing phase‐ and amplitude‐gated volumetric modulated arc therapy for stereotactic body radiation therapy using 3D printed lung phantom

**DOI:** 10.1002/acm2.12533

**Published:** 2019-01-22

**Authors:** Minsik Lee, KyoungJun Yoon, Byungchul Cho, Su Ssan Kim, Si Yeol Song, Eun Kyung Choi, SeungDo Ahn, Sang‐Wook Lee, JungWon Kwak

**Affiliations:** ^1^ Departments of Radiation Oncology Asan Medical Center University of Ulsan College of Medicine Seoul Republic of Korea

**Keywords:** 3D print, 4D lung phantom, amplitude‐gated VMAT, lung SBRT, phase‐gated VMAT

## Abstract

**Purpose:**

To compare the dosimetric impact and treatment delivery efficacy of phase‐gated volumetric modulated arc therapy (VMAT) vs amplitude‐gated VMAT for stereotactic body radiation therapy (SBRT) for lung cancer by using realistic three‐dimensional‐printed phantoms.

**Methods:**

Four patient‐specific moving lung phantoms that closely simulate the heterogeneity of lung tissue and breathing patterns were fabricated with four planning computed tomography (CT) images for lung SBRT cases. The phantoms were designed to be bisected for the measurement of two‐dimensional dose distributions by using EBT3 dosimetry film. The dosimetric accuracy of treatment under respiratory motion was analyzed with the gamma index (2%/1 mm) between the plan dose and film dose measured under phase‐ and amplitude‐gated VMAT. For the validation of the direct usage of the real‐time position management (RPM) data for respiratory motion, the relationship between the RPM signal and the diaphragm position was measured by four‐dimensional CT. By using data recorded during the beam delivery of both phase‐ and amplitude‐gated VMAT, the total time intervals were compared for each treatment mode.

**Results:**

Film dosimetry showed a 5.2 ± 4.2% difference of gamma passing rate (2%/1 mm) on average between the phase‐ vs amplitude‐gated VMAT [77.7% (72.7%–85.9%) for the phase mode and 82.9% (81.4%–86.2%) for the amplitude mode]. For delivery efficiency, frequent interruptions were observed during the phase‐gated VMAT, which stopped the beam delivery and required a certain amount of time before resuming the beam. This abnormality in phase‐gated VMAT caused a prolonged treatment delivery time of 366 s compared with 183 s for amplitude‐gated VMAT.

**Conclusions:**

Considering the dosimetric accuracy and delivery efficacy between the gating methods, amplitude mode is superior to phase mode for gated VMAT treatment.

## 1 INTRODUCTION

Respiratory‐induced movement is an important consideration during radiotherapy, particularly for volumetric modulated arc therapy (VMAT) for lung stereotactic body radiotherapy (SBRT). SBRT is a technique that is commonly used for treating early stage non‐small cell lung cancer and metastatic lung tumors.^1^, ^2^ Accordingly, the accuracy of dose delivery, conformity of dose distribution, and accurate target volume localization using motion management techniques are important for delivering safe and effective VMAT‐based SBRT.

There are various techniques for managing the respiratory‐induced motion of organs and tumors.^3^ Respiratory control approaches, including active breathing control and deep‐inspiration breath holding, not only increase the discomfort of a patient for an extended time but also alter the position of anatomical structures in the lung and diaphragm region.^4^

In gating approaches, the patient can breathe freely during the computed tomography (CT) scan and treatment. These techniques can be divided into two categories: phase gating and amplitude gating. In phase gating, the radiation beam is activated in a certain phase of the respiration cycle. In amplitude gating, the radiation beam is activated whenever a certain amplitude value is reached regardless of the phase in the patient's respiratory cycle. Amplitude gating is better at suppressing respiratory motion artifacts compared with phase gating.^5^ One study has shown that an irregular breathing pattern could be the reason for poor dosimetric results in phase‐gated studies, and it could be a more significant issue with phase gating compared with amplitude gating.^6^ In addition to the reasons mentioned above, amplitude gating may be more beneficial because amplitude gating has a shorter treatment time than phase gating. Short treatment times are highly recommended for dose‐delivery accuracy and delivery efficacy. Hoogeman et al.^7^ observed that the displacement of the patient exceeded 1 mm within a 3 min time span. Kim et al. reported an average translational difference in the target position of 1.8 ± 1.0 mm and a total rotational misalignment of up to 6° in 40 min.^8^ Similarly, Agazaryan et al.^9^ found patient movement of up to 3 mm along each axis within a 5 min time span, despite patient immobilization.

Nonetheless, most of the clinically used gating techniques are time‐based phase‐gating methods.^4^, ^10^, ^11^ Furthermore, several studies have compared the phase‐ and amplitude‐gating methods by acquiring the four‐dimensional (4D) CT image. To the best of our knowledge, no study has compared the dosimetric accuracy and delivery efficacy by generating a realistic patient‐specific phantom model.^12^, ^13^, ^14^

In this study, individualized lung phantoms were generated via a three‐dimensional printer (3D EDISON, Lokit, Korea) to achieve realistic simulation, and VMAT lung SBRT treatment was delivered. Gamma comparison was performed to evaluate the dosimetric accuracy of phase and amplitude gating, and the treatment time was compared by analyzing the trajectory log file to evaluate the treatment efficacy.

## 2 MATERIALS AND METHODS

**Figure 1:**
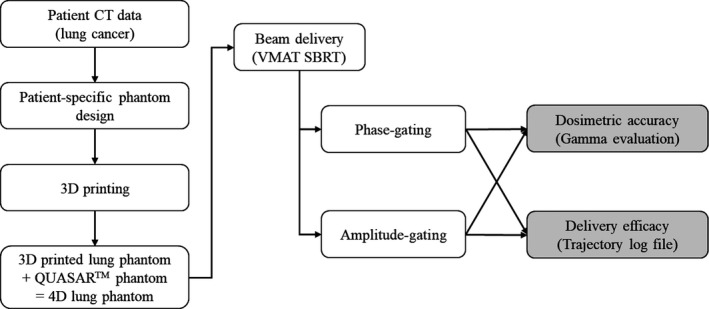
Simplified flowchart of the comparison process between the phase‐ and amplitude‐gated in dosimetric accuracy and the delivery efficacy.

Figure 1 shows the simplified flowchart of the comparison process between the phase‐ and amplitude‐gated in dosimetric accuracy and the delivery efficacy.

### 2.A Patient‐specific 3D‐printed respiratory lung phantoms

Four patients who had been treated by lung SBRT‐gated VMAT were selected for this study. We attempted to reproduce various lung and tumor conditions, and Table 1 shows the detailed information of each patient.

**Table 1:** Tumor volumes, respiratory‐tumor motion, and margin for four cases

Phantom no.	GTV (cc)		Location	LTV (cc)	Full motion (mm)	Margin (mm)	
	Patient	Phantom				ITV_GT_	ITV‐PTV
P1	4.4	4.6	RUL	305.7	12.4	1.8	5.0
P2	8.3	9.3	RUL	251.5	13.5	3.8	5.0
P3	17.9	18.6	RUL	186.9	14.6	3.6	5.0
P4	21.5	22.1	RLL	226.1	15.6	3.5	5.0

GTV: gross tumor volume; LTV: Lung tissue volume with the supporting structure of a 2 mm air gap omitting the tumor site in 3D‐printed patient lung phantom; ITV: internal target volume; GT: ground truth; PTV: planning target volume; RUL: right upper lobe; RLL: right lower lobe.

**Figure 2:**
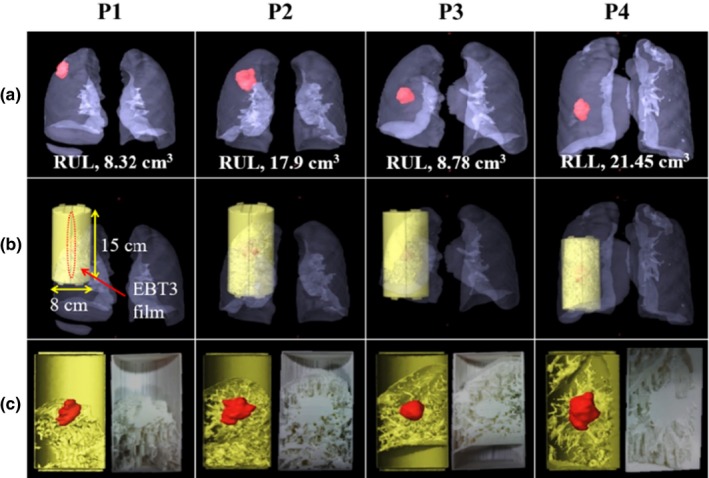
(a) GTV volume and tumor location of the four patient cases, (b) design of 3D‐printed lung phantom, (c) coronal sides of the rendering image (left), and the photographs of the fabricated 3D‐printed lung phantom (right).

Patient‐specific lung phantoms that closely simulated the actual lung tissue and respiratory patterns of each patient were generated using 3D‐printing techniques and respiratory breathing equipment. The 3D‐printed lung phantom was based on the end‐of‐exhale respiratory phase image from the 4D CT (LightSpeed RT16, GE healthcare, Chicago, IL) data using the Eclipse^™^ 10.0 (Varian Medical Systems, Palo Alto, CA) treatment planning system (TPS; Fig. 2). The 3D‐printed lung phantom had a cylindrical shape with 8 cm diameter, 15 cm length, and 2 mm surface thickness to enable it to be plugged into the hole of the QUASAR^™^ respiratory motion phantom (Modus Medical Devices, London, Canada). The respiratory motion phantom is designed to move cylindrical inserts in superior–inferior direction with various speed and amplitude according to the patient breathing pattern which acquired from the Real‐time Position Management (RPM; Varian medical systems, Palo Alto, CA, USA). The region representing the tumor was positioned at the center of the lung phantom and could be vertically bisected to measure the two‐dimensional (2D) dose distribution by inserting the EBT3 film in a coronal plane. We inserted the EBT3 film into the lung phantom only during the treatment beam was being delivered after the setup verification. The lung tissue of the phantom (excluding the tumor site) was produced with a mesh grid structure that was filled with 0.3 mm strips of 2 mm air gaps rather than an empty space to obtain a density similar to that of a real lung.^15^ Fused deposition modeling with a 3D printer was used for phantom generation, and polylactic acid with 1.25 g/cm^3^ density was used as the printing material.

### 2.B Data acquisition

**Figure 3:**
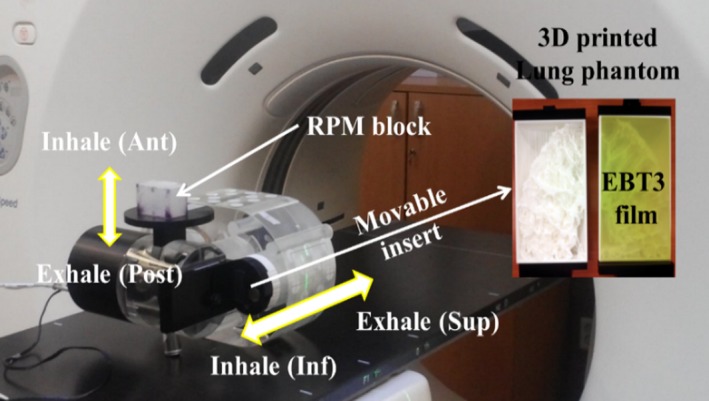
Installation of the 4DCT scan and the 4D lung phantom. The 4D lung phantom was composed of the QUASAR ^™^ phantom and 3D‐printed lung phantom. The EBT3 film was inserted inside the lung phantom.

Respiratory motion data for approximately 4 min were obtained via Varian's RPM system during a planned CT scan. It was continually repeated via QUASAR^™^ phantom for the beam delivery. The patient‐specific lung phantom was scanned using 4D CT with 1.25 mm slice thickness, and the RPM block was placed on the phantom (Fig. 3). The 4D CT images of each phantom with its respiratory pattern were reconstructed and sorted by phase‐ and amplitude‐gating methods. The same CT was used for both gating methods.

For phase gating, the breathing cycle was evenly divided into 10 phases, and a 30% to 70% gating window was selected as patient treatment. For amplitude gating, the lower and upper thresholds of the selected amplitude range were defined from the 40% to 50% gating window in the 10 phases used in phase gating of each patient.^16^ An element of visual and/or verbal coaching could be used to instruct a patient about the periodicity and amplitude of breathing to ensure treatment reproducibility.^17^ The choice of gating mode was influenced by several factors, including residual target motion within the selected period, the “duty cycle” or operational efficiency (ratio of beam delivery time to treatment time), lung tissue expansion, and breathing pattern stability.

VMAT plans were generated using the TPS system. Two‐arc gated VMAT plans with 6 MV photon beam (600 MU/min) were generated for four lung phantoms and delivered 5 Gy prescription dose to the planning target volume (PTV). The plan dose calculations were performed by Acuros^™^ XB with a 2.5 mm grid. The plans were delivered on a TrueBeam^™^ 1.5 linear accelerator (LINAC) (Varian Medical Systems, Palo Alto, CA) with a multileaf collimator (MLC) and Varian's RPM system for the respiratory gating. Prior to beam delivery, image‐guided radiation therapy (IGRT) procedures, such as those in clinical practice, were performed to reduce setup errors in 4D phantom because of patient breathing by using the 3D matching of 2D fluoroscopic images and cone‐beam CT images. The 3D matching carried out by setting up based on the surface of the phantom initially, and internal structures were aligned. Similar to that in the 4D CT procedure, two treatment modes (phase‐ and amplitude‐gated VMAT) were used for beam delivery. All other conditions were the same except for the gating method.

### 2.C Analysis

To assess the dosimetric accuracy of phase‐ and amplitude‐gated VMAT treatment, gamma analysis was performed using EBT3 film under the 2%/1 mm criterion with 80% passing rate. Many studies recommended a stricter gamma criterion of 2%/1 mm or 2%/2 mm rather than 3%/3 mm, particularly for VMAT quality assurance (QA). However, 3%/3 mm was still an acceptable standard for evaluating IMRT and other plans in a clinical setting.^18^, ^19^, ^20^, ^21^ The film images were scanned in 48‐bit RGB (red, green, and blue) mode with 72 dpi resolution (pixel size: 0.35 mm) and were measured and analyzed using FilmQA pro 2012 software (Ashland Inc., Bridgewater, NJ, USA). Delivery efficacy was also evaluated by analyzing the trajectory log file from the treatment records. The TrueBeam^™^ control system generated a trajectory log file, which recorded the beam status data and various information about the expected and actual values of the treatment. By using this information, the following were evaluated: total treatment time, total time interval for the treatment, gate‐on time, time interval when the gating system allowed the beam on, beam‐on time, and time interval when the beam was delivered.

## 3 RESULTS

### 3.A Dosimetric accuracy

Table 2 shows the results of gamma analysis under the 2%/1 mm criterion according to the phase‐ and amplitude‐gating methods with patient‐specific lung phantoms. There were noticeable differences in gamma passing rate according to the gating method. On average, the gamma passing rate of the amplitude gating was 5.2 ± 4.2% higher than the phase gating. Furthermore, it exceeded 80% in all cases, which was an acceptable passing rate under the 2%/1 mm criterion. On the contrary, every case in phase gating, except for P4, did not exceed 80%. The 3%/3 mm criterion, which was generally recommended and routinely applied in clinical practice, showed similar average passing rates on both gating strategies (phase: 98.3; amplitude: 98.7).

**Table 2:** Gamma evaluation results of the phase‐ and amplitude‐gating methods with 2%/1 mm criterion

Phantom no.	Gamma passing rate (2%/1 mm)		
	Phase	Amplitude	|Δ*γ*|
P1	73.8	82.5	8.7
P2	78.2	81.4	3.2
P3	72.7	81.3	8.6
P4	85.9	86.2	0.3
Mean ± SD	77.7 ± 6.0	82.9 ± 2.3	5.2 ± 4.2

### 3.B Delivery efficacy

**Figure 4:**
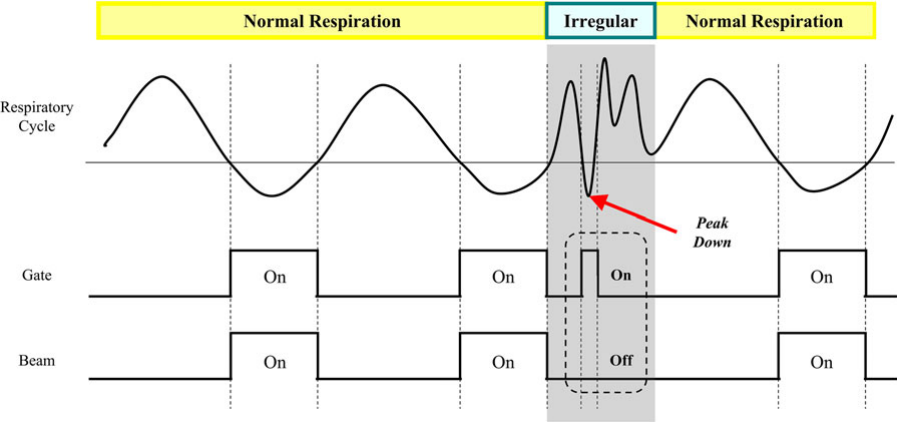
Schematic illustration of the phase‐gated beam delivery with an irregular breathing pattern (beam interruption). The shaded area shows the moment that the beam was off when the gating was on.

In the phase‐gating method shown in Fig. 4, gating‐ and beam‐on intervals could not be synchronized owing to an irregular breathing pattern with an unexpected sudden change (marked as beam interruption). When this phenomenon occurred, the system automatically shut down until the periodicity of the normal breathing pattern was reestablished. There were also moments when the beam instantly turned off even without a sudden irregular change of breathing pattern. In the four patient‐specific phantoms, this type of beam interruption occurred 39 times on average in phase gating (49, 63, 29, and 17) but rarely occurred in amplitude gating (1, 0, 1, and 0). As the beam‐off time and frequency increased, the treatment efficacy decreased, and the overall treatment time was extended.

**Figure 5:**
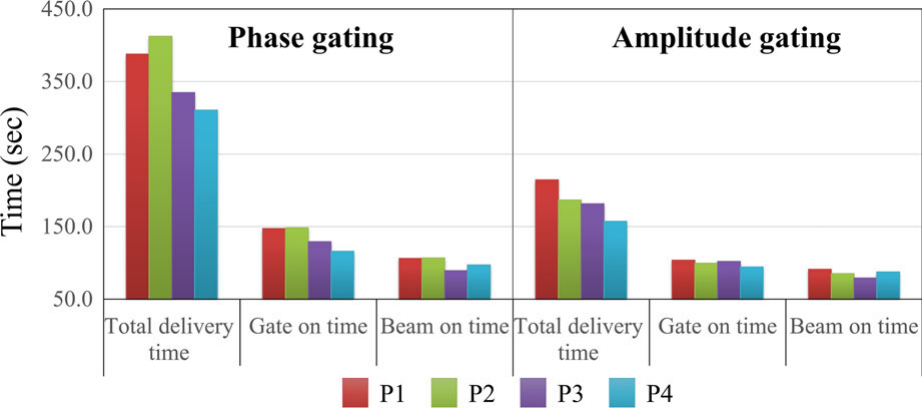
Graph to compare the treatment time between phase‐ and amplitude‐gating methods.

Figure 5 shows the total delivery time, gate‐on time, and beam‐on time according to the gating method. As expected, the total delivery time of the phase‐gating method was approximately two times longer than that of the amplitude‐gating method. Phase gating took approximately 6 min (361 ± 46 s), whereas amplitude gating took approximately 3 min (185 ± 24 s). Even the largest difference of total delivery time between the gating methods was 225 s (2.2 times) at P2. Likewise, gate‐on time and beam‐on time in amplitude gating were approximately 1.3 times (100 and 135 s) and 1.2 times (86 and 99 s) faster than those in phase gating, respectively. Table 3 shows the summary of phase‐ and amplitude‐gated VMAT deliveries, including the number of beam interruptions. According to this table, the average numbers of beam interruptions for the phase‐ and amplitude‐gating methods were 39.5 and 0.5, respectively.

**Table 3:** Summary of phase‐ and amplitude‐gated VMAT deliveries

Gating method	Phantom no.	Total delivery (s)	Gate on (s)	Beam on (s)	Interruption (#)
Phase	P1	387	148	106	49
	P2	412	148	107	63
	P3	335	129	89	29
	P4	311	116	97	17
	Mean ± SD	361 ± 46	135 ± 16	99 ± 8	39.5
Amplitude	P1	215	104	91	1
	P2	187	100	85	0
	P3	182	102	79	1
	P4	157	94	88	0
	Mean ± SD	185 ± 24	100 ± 4	86 ± 5	0.5

## 4 DISCUSSION

The QA of gating systems should include an analysis of both the time delay and dosimetric characteristics of the gated delivery. Thus, we compared the dosimetric impact and treatment delivery efficacy of phase‐ and amplitude‐gated VMAT for stereotactic lung cancer treatment by using realistic patient‐specific lung phantoms fabricated with a 3D printer. According to the results, there are noticeable differences between the two gating methods in terms of dosimetric accuracy. The average gamma passing rate of the amplitude gating was 5.2 ± 4.2% higher than the phase gating and in all cases exceeded 80%, which was an acceptable passing rate at the 2%/1 mm criterion. Similar to the other studies, these results support that amplitude gating is better in terms of dosimetric impact.^5^, ^6^ Amplitude gating was also better in terms of delivery efficacy because phase gating takes double the total delivery time compared with amplitude gating on average. We think that not increasing in total treatment time in amplitude gating is a major clinical benefit. The longer the treatment time, the possibility for the patient motion will be increased and the accuracy of treatment will be decreased accordingly. It is assumed that this time difference is mainly due to the moment that the beam instantly turns off even without a sudden irregular change of breathing pattern (i.e., beam interruption). This phenomenon occurs more frequently in phase gating (~39.5 times) than in amplitude gating (~0.5 times). Gated VMAT radiotherapy is periodically interrupted as a response to a gating signal from the RPM system. It involves complex controls such as MLC, dose rate, and gantry rotation. In the actual delivery of gated VMAT, beam interruption involves the slowing down of the gantry to a complete stop followed by the reverse rotation of the gantry until it reaches the position right before the gate‐off signal is triggered. This phenomenon has been observed in many studies. Qian et al.^22^ demonstrated that beam interruption could further increase gated VMAT treatment time from a few percent to 25%, and it was highly dependent on the patient's respiratory pattern. Oh et al.^23^ and Heo et al.^24^ demonstrated that a small number of beam interruptions were clinically acceptable in VMAT delivery and show only minimal changes in gamma passing rate, dose volume parameters, and log file analysis. In a slightly different case, Inaniwa et al.^25^ suggested that the curative dose could increase by 20% or more compared with the planned dose if the interruption time extended to 30 min or longer in carbon‐ion radiotherapy, and it should be considered if a longer dose‐delivery procedure time was anticipated. Beam interruption is known to be caused by the gating parameters and by sudden movements of the patient, such as a cough.^24^ An insufficient correlation between the external surrogate marker and the internal tumor motion can be another reason for an interruption. However, it is suspected that patient motion may not be the primary cause of beam interruption because it also occurs in phantom experiments with phase gating, wherein unpredictable motion cannot occur. The same phenomenon has been observed with the recently installed VitalBeam^™^ 2.5 LINAC (Varian Medical Systems, Palo Alto, CA) at our site, and further research is being planned for cause analysis.

A further point to consider is that the respiration patterns of the four patients selected for the test are not particularly abnormal. Considering the clinical risk of a rapid shift of the baseline, it would be better to use the phase‐gating method in most cases. On the contrary, if the respiratory pattern is stable without a baseline shift, particularly if the inhale is irregular and short but the exhale is long, amplitude gating will be advantageous in terms of treatment time. Considering that baseline shift is common in clinical settings, including experiments with patients who have irregular or extreme breathing patterns, it may be more effective to analyze the performance of each gating method. Additional experiments are planned by selecting patients with irregular breathing to compare the results according to the gating method.

## 5 CONCLUSIONS

We investigated the dosimetric impact and treatment delivery efficacy of phase‐gated VMAT vs amplitude‐gated VMAT for stereotactic lung cancer treatment by using realistic lung phantoms fabricated with a 3D printer. Patient‐specific lung phantoms that closely simulated the actual lung tissue were generated using 3D printing techniques, and the respiratory patterns of each patient were demonstrated with the QUASAR^™^ breathing equipment.

Considering the two aspects (dosimetric impact and treatment delivery efficacy) of the phase‐ and amplitude‐gating strategies, amplitude mode would be superior to phase mode for gated VMAT treatment. The amplitude method shows a 5.2 ± 4.2% higher average gamma passing rate than the phase method under the 2%/1 mm criterion, but it takes approximately one‐half the total treatment time. This time difference is mainly due to the beam interruptions and this phenomenon occurs more frequently in phase gating (~39.5 times) than in amplitude gating (~0.5 times). Although the exact reasons are not yet known, there are many studies reporting this phenomenon and therefore additional experiments are being planned for cause analysis. Our study can be a good reference for the application of amplitude gating in the treatment of gated VMAT for lung SBRT.

## CONFLICT OF INTEREST

No conflicts of interest.
